# Supplementary material for: Loss of testosterone impairs anti-tumor neutrophil function
Source: Nat Commun. 2020 Mar 31;11:1613. doi: 10.1038/s41467-020-15397-4 (PMC7109066; doi:10.1038/s41467-020-15397-4)
Supplement: Supplementary file 1 — Supplementary Information [file 41467_2020_15397_MOESM1_ESM.pdf]

Loss of testosterone impairs anti-tumor neutrophil function

Markman, et. al.

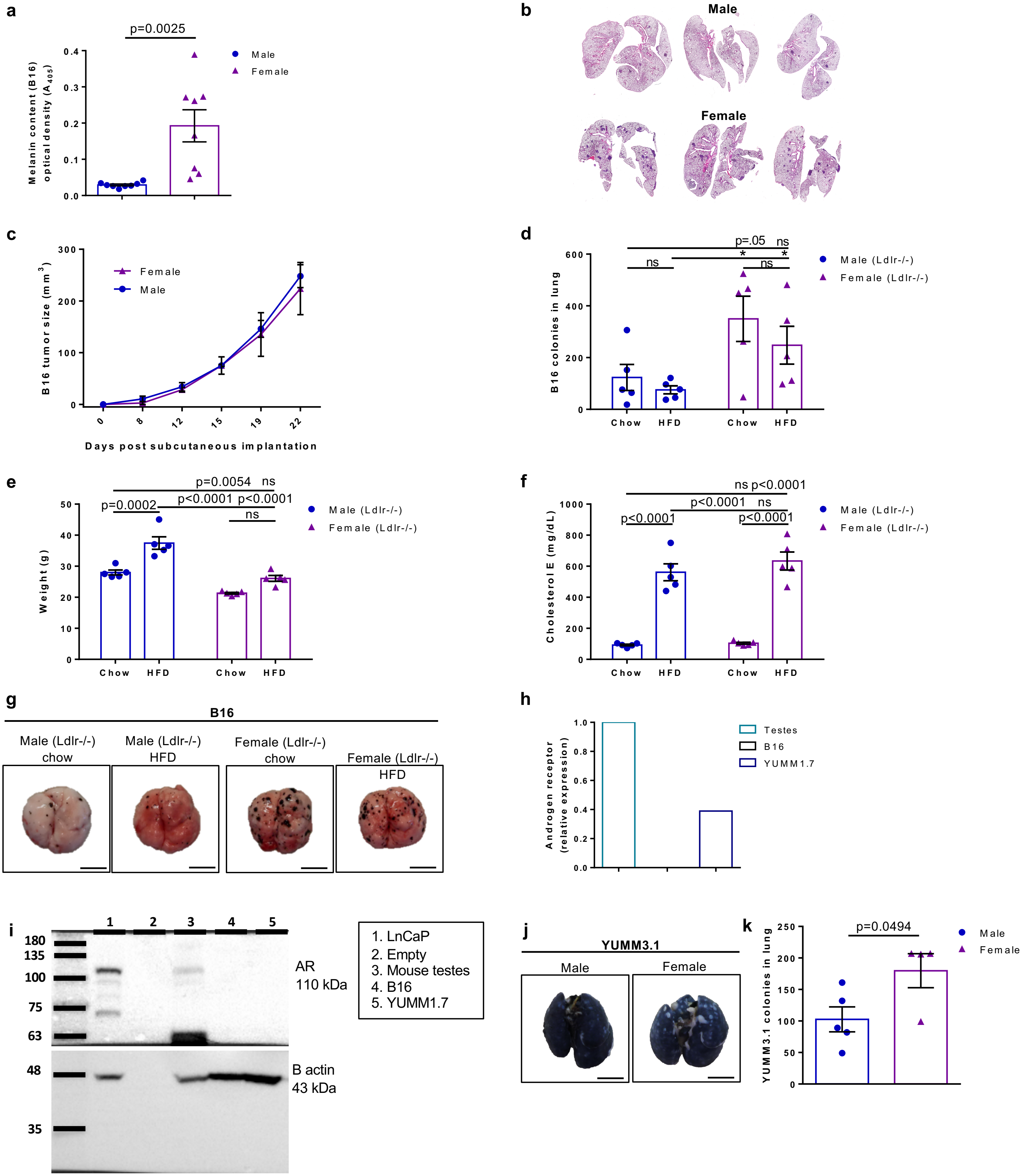

Supplementary Figure 1. Melanoma tumor burden in the lungs of female and castrated male mice is higher than in sham male mice in experimental metastatic models. (**a**) Measurement of melanin content from the left lobe of the lung B16-tumor bearing C57Bl/6 mice (n=8 per group). (**b**) Representative hematoxylin and eosin (H&E) staining of lungs from B16-bearing male and female mice. (**c**) Tumor volume of B16 tumors subcutaneously implanted in male and female mice (n=5 per group). (**d**) Number of tumor colonies, (**e**) body weight, (**f**) cholesterol E levels, and (**g**) representative images of B16-tumor bearing male and female *Ldlr* KO mice on normal chow or high fat diet (HFD; n=5 per group). (**h**) Relative expression of the androgen receptor in B16 and YUMM1.7 cells to mouse testes. (**i**) Western blot results of AR protein in cell lines and mouse testes (positive control) using beta actin as a loading control. (**j**) Representative lung images and (**k**) number of YUMM3.1 tumor colonies in male and female mice (n=5 male, n=4 female). Data in **a** and **c-h, k** are mean ± s.e.m. by two-tailed unpaired t test (**a, k**), repeated measures t test (**e**) and 1-way ANOVA with Bonferoni post-tests analysis (**d-f**). Data in **a-b** are representative of at least 3 independent experiments; data in **e**, **j-k** are representative of 2 independent experiments. The scale bar represents 0.5cm for images of lung.

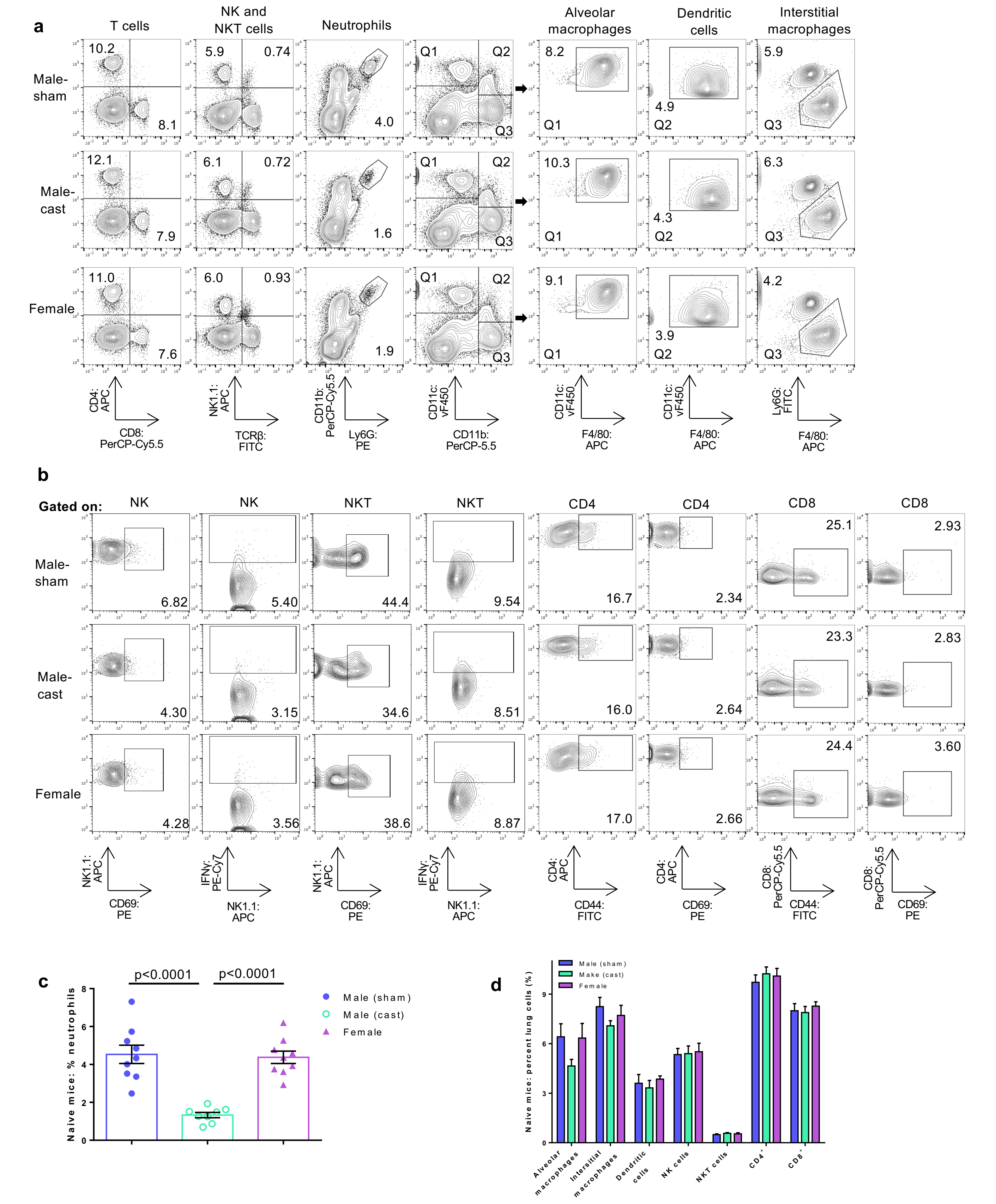

Supplementary Figure 2. Sham male mice have increased neutrophil infiltration and NK cell activation in the lungs of melanoma injected mice. (**a**) Representative flow cytometry gates for percent cells in the lungs of male, castrated, and female C57Bl/6 B16-tumor bearing mice for the following cell types: $CD4^+$ T cells, $CD8^+$ T cells, NK cells, NKT cells, $CD11b^+Ly6G^+$ neutrophils, $CD11b^-$ $CD11c^+$ $F4/80^+$ alveolar macrophages, $CD11b^+$ $CD11c^+$ $F4/80^+$ dendritic cells, and $CD11b^+$ $CD11c^-$ $F4/80^+$ interstitial macrophages. (**b**) Representative flow cytometry gates for percent activated cells in the lungs of male, castrated, and female C57Bl/6 for: $CD69^+$ NK cells, $IFN\gamma^+$ NK cells, $CD69^+$ NKT cells, $IFN\gamma^+$ NKT cells, $CD4^+CD44^+$ T cells, $CD4^+CD69^+$ T cells, $CD8^+CD44^+$ T cells, and $CD8^+CD69^+$ T cells. (**c**) Flow cytometry analysis of lung-infiltrating $CD11b^+$ $Ly6G^+$ neutrophils and (**d**) additional cells types in naïve mice. Data in **c-d** are mean ± s.e.m. analyzed by 1-way ANOVA with Bonferoni post-tests analysis. Data in **c-d** are representative of 2 independent experiments (n=8-9 per group).

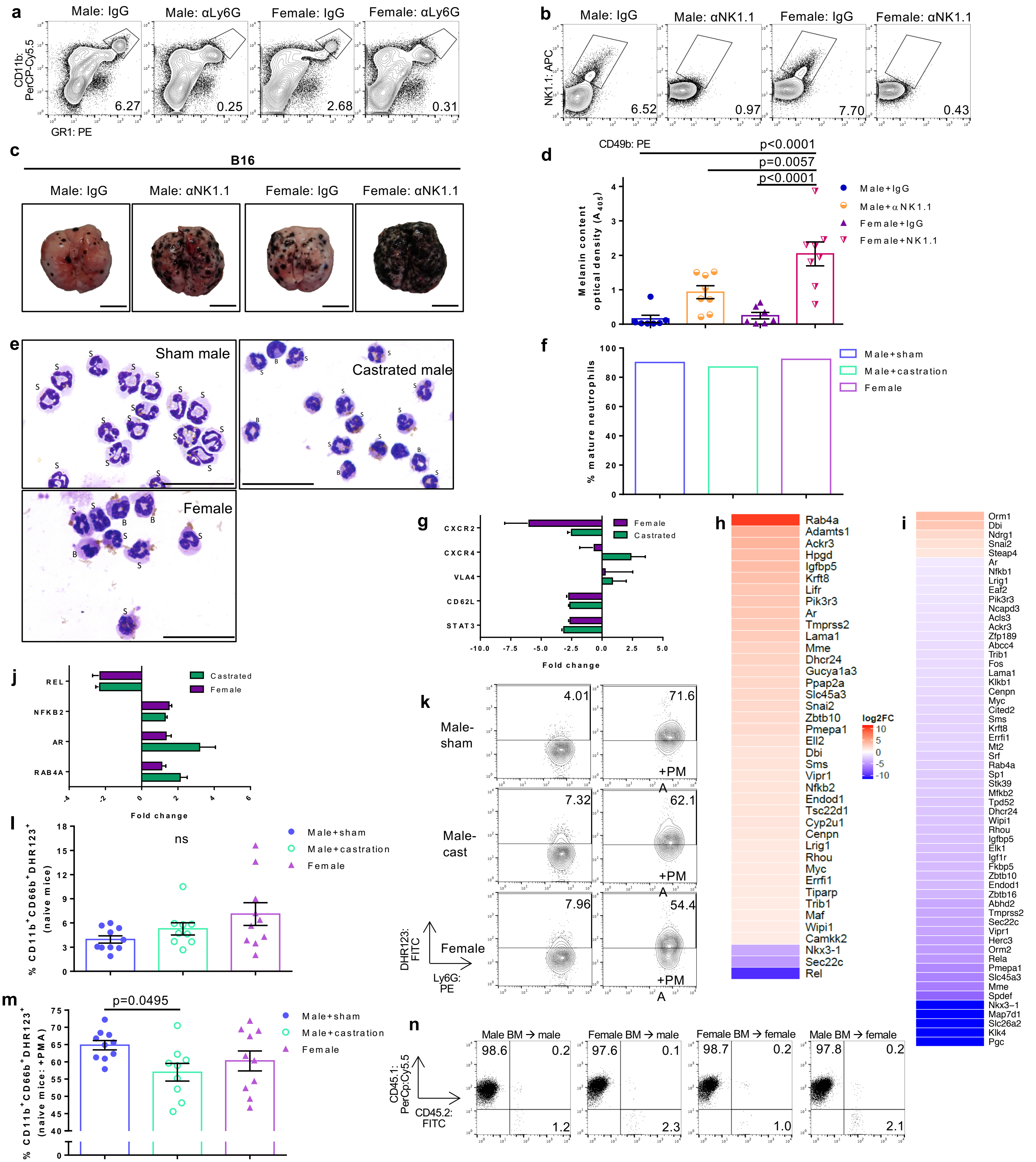

Supplementary Figure 3. Neutrophils from sham male tumor bearing mice have an anti-tumor effect. Flow cytometry gates for (**a**) neutrophils or (**b**) NK cells following depletion with anti-Ly6G (1A8) or anti-NK1.1 (PK136) mAbs, respectively. (**c**) Representative images and (**d**) melanin content from the left lobe of the lungs of B16-tumor bearing C57Bl/6 mice following control IgG or NK cell depletion with an anti-NK (PK136) mAb (n=7 IgG groups, n=8 αNK1.1 groups). (**e**) Representative images of isolated neutrophils from the lungs of sham male, castrated male, and female B16-tumor bearing mice. B=banded, immature neutrophil; S=segmented, mature neutrophil. (**f**) Percent of lung neutrophils from naive mice that have a mature, segmented appearance morphologically (n=5 per group; pooled). (**g**) qPCR results for select markers; fold change of isolated BM-derived neutrophils from tumor bearing-castrated or female mice compared with sham male mice (n=15; 3 pooled sets of 5 from 3 independent experiments; mean+s.e.m.). (**h**) qPCR AR pathway array data comparing lung-derived neutrophils (h) or prostates (i) from castrated mice to sham operated male mice with at least a 2-fold difference (pooled n of 5-6). (**j**) Validation of select qPCR array data for neutrophils (n=15; 3 pooled sets of 5 from 3 independent experiments; mean+s.e.m.) by qPCR. (**k**) Representative flow cytometry gates of DHR123⁺ unstimulated and PMA-stimulated neutrophils from sham male, castrated male, and female mice. Percent of lung neutrophils from naïve mice producing DHR123 without stimulation (**l**) or with PMA stimulation (**m**) (n=10 male+sham, n=9 male+castration, n=10 female). (**n**) Representative flow cytometry gates for confirmation of efficient bone marrow (BM) reconstitution. Data in **d**,**l**, and **m** are mean ± s.e.m. analyzed by 1-way ANOVA with Bonferoni post-tests analysis or (**f**) z-score by difference of two proportions. Data in **c-d** are representative of 2 independent experiments and **e, l-m** of 3 representative experiments. The scale bar represents 0.5cm for images of lung and 50 μM for microscope images.

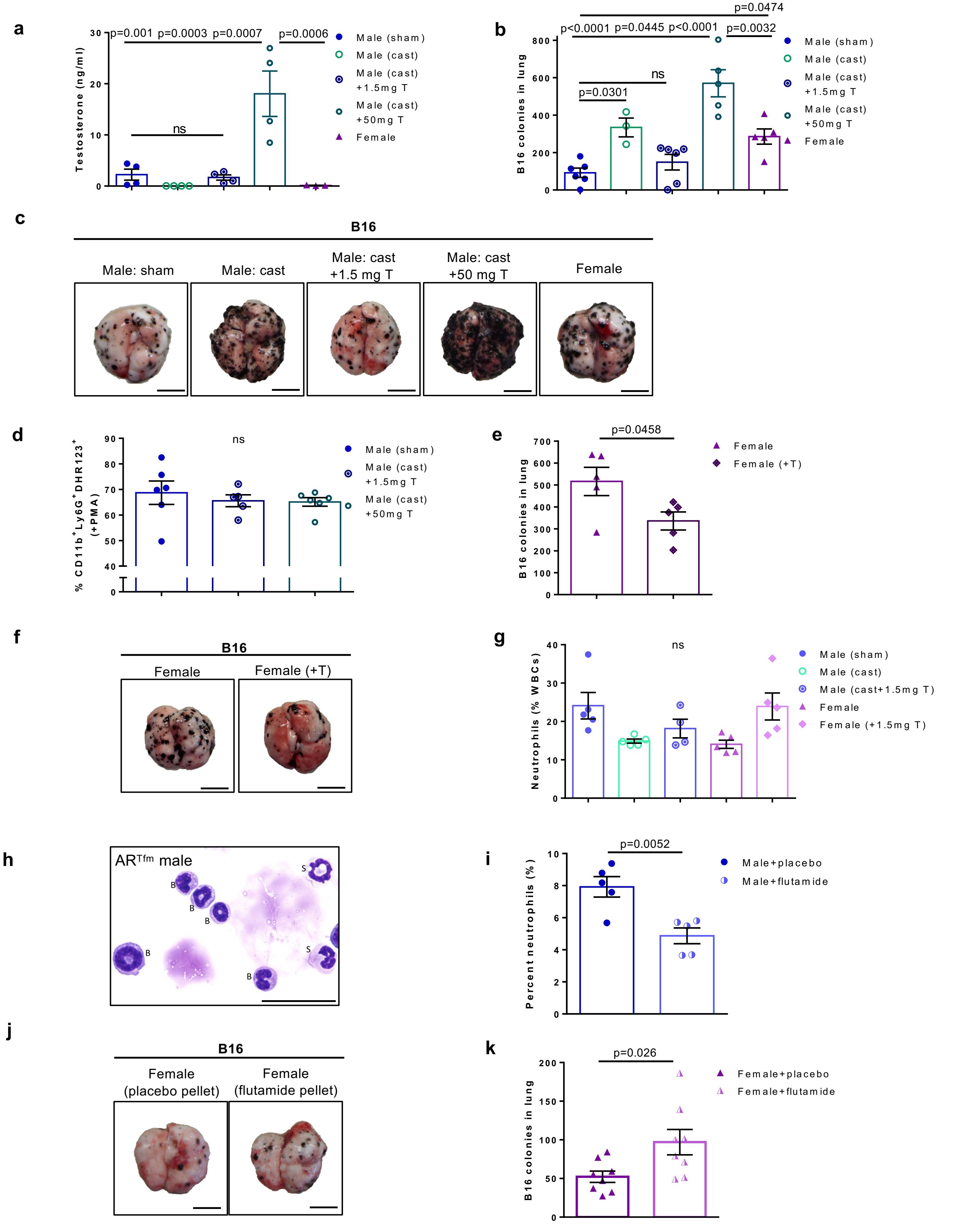

Supplementary Figure 4. Testosterone signaling in immune cells impacts tumor burden. (**a**) Circulating testosterone levels in serum (n=4 per male group, n=3 female mice). (**b**) B16 tumor burden and (**c**) representative images of the lungs of mice following castration and testosterone supplementation at the time of castration at two concentrations using 60-day slow release pellets (n=6 male(sham), n=3 male(cast), n=6 (male(cast)+1.5mg T, n=5 male(cast)+50mg T and female). (**d**) Percent of PMA stimulated DHR123⁺ lung neutrophils from B16-tumor bearing mice following castration and testosterone supplementation (n=6 male(sham), n=5 (male(cast)+1.5mg T, n=6 male(cast)+50mg T). (**e**) B16 tumor burden and (**f**) representative lung images from female mice implanted with 1.5 mg testosterone 60-day slow release pellets (n=5 per group). (**g**) Neutrophil percent of WBCs of naïve mice determined by complete blood count (CBC; n=5 per group except n=4 male(cast)+1.5mg T). (**h**) Representative image of isolated neutrophils from the lungs of naïve AR^{Tfm} mice. B=banded, immature neutrophil; S=segmented, mature neutrophil. (**i**) Percentage of neutrophils in lungs of male mice implanted with either a placebo or flutamide pellet (n=5 per group). (**j**) representative images of the lungs and (**k**) B16 tumor burden of female mice following implantation of a placebo or slow-release flutamide pellet (n=8 per group). Data in **a-b**, **d-e, g** and **i** are mean ± s.e.m. analyzed by 1-way ANOVA with Bonferoni post-tests analysis (**a-b, d, g**), or by two-tailed unpaired t test (**e, i, k**). Data in **b-d** and **h-k** are representative of at least 2 independent experiments. The scale bar represents 0.5cm for images of lung and 50 µM for microscope images.

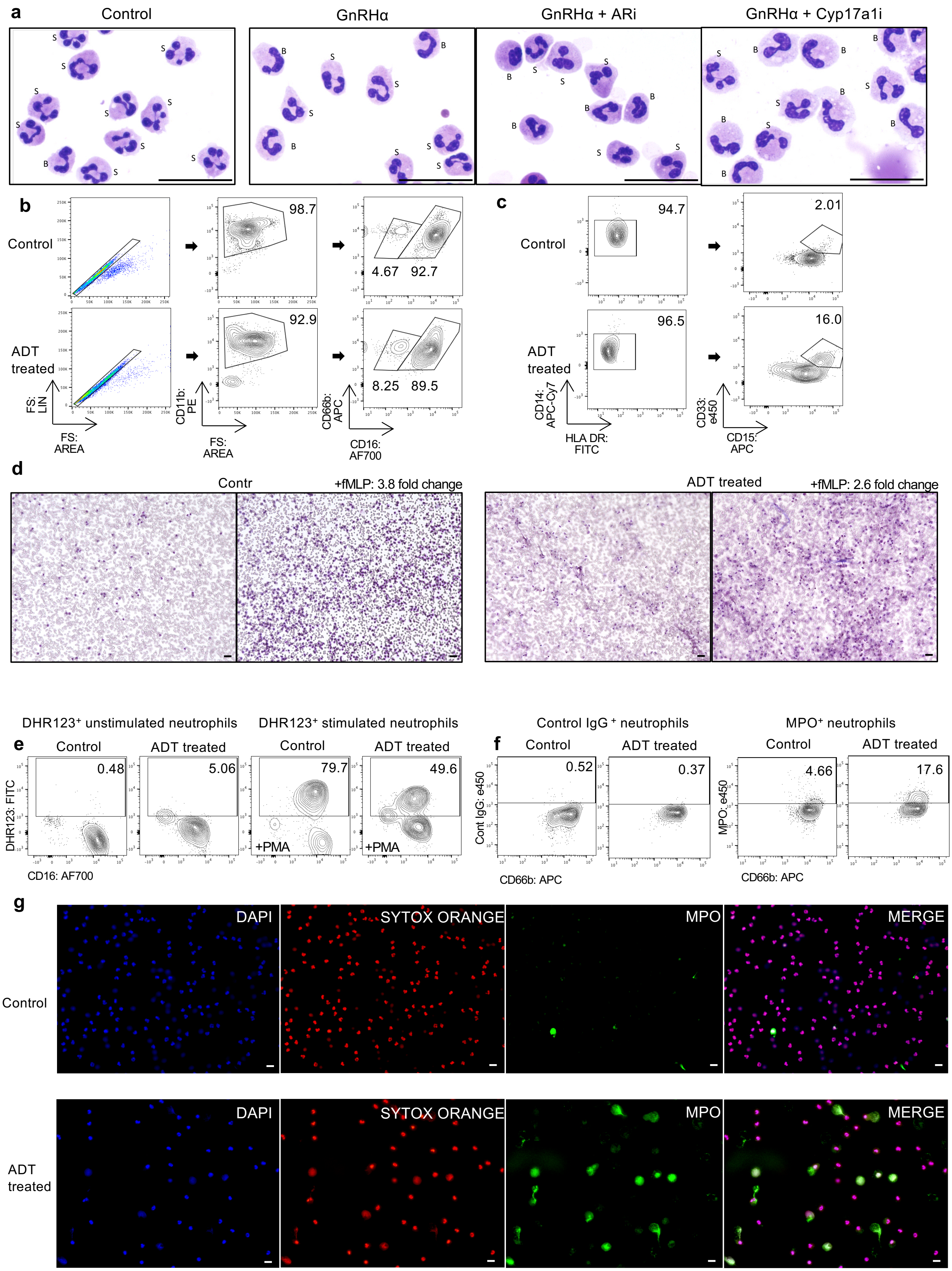

Supplementary Figure 5. Analysis of neutrophils from control or androgen deprivation therapy (ADT) treated patients. (**a**) Representative images of isolated neutrophils from the circulation of prostate cancer patients not receiving any treatment, or different forms of androgen ablation therapy (ADT). B=banded, immature neutrophil; S=segmented, mature neutrophil. Representative flow cytometry gating strategies for (**b**) CD16 expression and (**c**) MDSC-like markers. (**d**) Representative image of control versus chemoattractant (fMLP) containing transwells, 20x. Representative flow cytometry gates for (**e**) DHR123⁺ unstimulated and stimulated neutrophils and (**f**) MPO expression. (**g**) Representative NET staining, 40x. The scale bar represents 50 µM.

Patient	Prostatectomy	Disease status	Current treatment
1	yes	recurrent	none
2	yes	undetectable	none
3	yes	recurrent	none
4	yes	recurrent	none
5	yes	recurrent	none
6	no	active surveillance	none
7	yes	recurrent	none
8	yes	non-recurrent	none
9	no	undetectable	leuprolide
10	no	unknown	leuprolide
11	yes	recurrent	leuprolide
12	yes	recurrent	leuprolide
13	no	active	leuprolide
14	yes	recurrent	leuprolide
15	yes	recurrent	leuprolide
16	yes	recurrent	leuprolide
17	yes	recurrent; no active	triptorelin, enzalutamide
18	yes	recurrent	leuprolide, enzalutamide
19	yes	recurrent; no active	leuprolide, bicalutamide
20	no	recurrent	leuprolide, bicalutamide
21	yes	recurrent; no active	leuprolide, bicalutamide
22	no	active	leuprolide, bicalutamide
23	no	recurrent	leuprolide, apalutamide
24	no	active	leuprolide, abiraterone
25	no	recurrent	leuprolide, abiraterone
26	no	recurrent; no active	leuprolide, abiraterone
27	no	recurrent	goserelin, abiraterone
28	yes	recurrent; no active	leuprolide, abiraterone
29	no	no active	leuprolide, abiraterone
30	no	active	degarelix, abiraterone

Supplementary Table 1. Patients’ baseline characteristics.
