# Supplementary material for: Loss of testosterone impairs anti-tumor neutrophil function
Source: Nat Commun. 2020 Mar 31;11:1613. doi: 10.1038/s41467-020-15397-4 (PMC7109066; doi:10.1038/s41467-020-15397-4)
Supplement: Supplementary file 3 — Reporting Summary [file 41467_2020_15397_MOESM3_ESM.pdf]

nature research

Corresponding author(s): Janet L. Markman, Rebecca A. Porritt, Daiko Wakita, Malcolm E. Lane , Magali Noval Rivas, Michael Luu, Edwin M Posadas, Timothy R. Crother, and Moshe Arditi

Last updated by author(s): Feb 23, 2020

Reporting Summary

Nature Research wishes to improve the reproducibility of the work that we publish. This form provides structure for consistency and transparency in reporting. For further information on Nature Research policies, see Authors & Referees and the Editorial Policy Checklist .

Statistics

For all statistical analyses, confirm that the following items are present in the figure legend, table legend, main text, or Methods section.

n/a	Confirmed	
☐	☒	The exact sample size (*n*) for each experimental group/condition, given as a discrete number and unit of measurement
☒	☐	A statement on whether measurements were taken from distinct samples or whether the same sample was measured repeatedly
☐	☒	The statistical test(s) used AND whether they are one- or two-sided<br>*Only common tests should be described solely by name; describe more complex techniques in the Methods section.*
☒	☐	A description of all covariates tested
☐	☒	A description of any assumptions or corrections, such as tests of normality and adjustment for multiple comparisons
☐	☒	A full description of the statistical parameters including central tendency (e.g. means) or other basic estimates (e.g. regression coefficient) AND variation (e.g. standard deviation) or associated estimates of uncertainty (e.g. confidence intervals)
☒	☐	For null hypothesis testing, the test statistic (e.g. *F*, *t*, *r*) with confidence intervals, effect sizes, degrees of freedom and *P* value noted<br>*Give P values as exact values whenever suitable.*
☒	☐	For Bayesian analysis, information on the choice of priors and Markov chain Monte Carlo settings
☒	☐	For hierarchical and complex designs, identification of the appropriate level for tests and full reporting of outcomes
☒	☐	Estimates of effect sizes (e.g. Cohen's *d*, Pearson's *r*), indicating how they were calculated

Our web collection on statistics for biologists contains articles on many of the points above.

Software and code

Policy information about availability of computer code

Data collection	LSRII BD FACS Diva 8 (BD Biosciences), Keyence Biorevo B-viewer, SpectraMax Pro 5.4
Data analysis	Keyence Biorevo B-analyzer, Flowjo version 10.5, GraphPad Prism 6.0, (Excel, Office 365), GIMP, ImageLab 5.2.1, Biorad CFX-manager 3.1,

For manuscripts utilizing custom algorithms or software that are central to the research but not yet described in published literature, software must be made available to editors/reviewers. We strongly encourage code deposition in a community repository (e.g. GitHub). See the Nature Research guidelines for submitting code & software for further information.

Data

Policy information about availability of data

All manuscripts must include a data availability statement. This statement should provide the following information, where applicable:

- Accession codes, unique identifiers, or web links for publicly available datasets
- A list of figures that have associated raw data
- A description of any restrictions on data availability

The authors declare that the data supporting the findings of this study are available within the paper and its Supplementary Information.

Field-specific reporting

Please select the one below that is the best fit for your research. If you are not sure, read the appropriate sections before making your selection.

☒ Life sciences ☐ Behavioural & social sciences ☐ Ecological, evolutionary & environmental sciences

Life sciences study design

All studies must disclose on these points even when the disclosure is negative.

Sample size	The sample sizes in each experimental group was determined from our preliminary experiments and based on 80% power and two- sided tests for 5% level of significance. Animal experiments used a minimum of 5 mice per group.
Data exclusions	All samples that met adequate experimental conditions were included in the analysis. Samples that failed the Grubb's test for outliers in GraphPad 6.0 were excluded.
Replication	Experiments were successfully performed a minimum of two times and/or with sufficient animals per group to demonstrate statistical significance.
Randomization	The experiments were not randomized. Surgeries were performed on the same day, sham surgeries were performed on control groups, the same batch and number of cancer cells were injected per experiment, and all animals were housed under the same conditions.
Blinding	The human data was blinded using a unique patient identifier. For animal experiments, blinding was not performed as the sex of the mouse or evidence of castration are readily apparent and is not ethical to mix cages of male and female, or castrated and sham mice. All analysis was performed consistently during all studies and all tumor counts were performed by the same investigator.

Reporting for specific materials, systems and methods

We require information from authors about some types of materials, experimental systems and methods used in many studies. Here, indicate whether each material, system or method listed is relevant to your study. If you are not sure if a list item applies to your research, read the appropriate section before selecting a response.

Materials & experimental systems

n/a	Involved in the study	
☐	☒	Antibodies
☐	☒	Eukaryotic cell lines
☒	☐	Palaeontology
☐	☒	Animals and other organisms
☐	☒	Human research participants
☒	☐	Clinical data

Methods

n/a	Involved in the study	
☒	☐	ChIP-seq
☐	☒	Flow cytometry
☒	☐	MRI-based neuroimaging

Antibodies

Antibodies used

Antibodies were used for flow cytometry, immunofluoresecent tissue staining and cell depletion.

- Flow cytometry and cell sorting :

Mouse: FcBlock (CD16/CD32, Clone 2.4G2; 70-0161-M001; Tonbo Biosciences); Ly6G (1A8; PE; BioLegend, 127608) or Ly6G (Gr-1; PE; eBioscience, 12-5931-82); CD11b (M1/70; PerCP-Cy5.5; Tonbo Biosciences, 35-0112) and F4/80 (BM8.1; APC; Tonbo Biosciences, 20-4801); CD11b and CD11c (N418; vF450; Tonbo Biosciences, 75-0114); CD4 (RM4-5; APC; Tonbo Biosciences, 35-0042); CD8a (Ly-2; PerCP-Cy5.5; eBioscience, 15-0081-82); NK1.1(PK136; APC; eBioscience, 17-5941-82) or CD49b (DX5; PE; eBioscience); CD69 (H1.2F3; PE; eBioscience, 12-0691-83); CD45.1 (A20; PerCp-Cy5.5; eBioscience, 45-0453-80) and CD45.2 (104; FITC; Tonbo Biosciences, 35-0454)

Human: FcR blocking antibody (130-059-901, MACS Miltenyi Biotec); Cd11b (ICFR44; PE, Tonbo Biosciences, 50-0118-T100), CD14 (HCD14;APC/Cy7, BioLegend, 325620), CD15 (MMA; APC, eBioscience, 17-0158-42), CD16 (CB16; Alexa Fluor 700, eBioscience, 56-0168-42), CD33 (P67.6; eFluor 450, eBioscience, 48-0337-42), CD45 (HI30; violetFluor 450, Tonbo Biosciences, 75-0459-T100), CD66b (G10F5; APC, eBioscience, 17-0666-42), HLA-DR (L243; FITC, Tonbo Biosciences 35-9952-T100), Mouse IgG1 K IsoControl (P3.6.2.8.1; eFluor 450, eBioscience, 48-4714-82), and MPO (MPO455-8E6; eFluor 450, eBioscience, 48-1299-42).

Mouse and human: dihydrorhodamine 123 (DHR123, CAS 109244-58-8; Santa Cruz Biotechnology)

- Immunofluorescent cell staining: SYTOX orange (ThermoFisher Scientific, S11368) and MPO (Abcam, ab9535)

-Slide staining: donkey-anti-rat IgG (AlexaFluor594, Thermofisher Scientific, A-21209)

- Cell depletion: Ly6G monoclonal antibody (mAb) (Clone 1A8, BP0075, Bio X Cell); control rat IgG (14131, Sigma); NK1.1 mAb (Clone PK136, BE0036, Bio X Cell); control mouse IgG (I5381, Sigma-Aldrich).

Western Blot: Anti-AR (Santa Cruz Biotechnology, catalog sc-7305), anti-β-Actin (Sigma, catalog A5316), and HRP-conjugated goat anti-mouse (Jackson ImmunoResearch, catalog 115-035-003).

Validation	Pre-validated antibodies were purchased from well recognized vendors and were reported by other researchers. We based specificity on their provided description and data sheets and ensured that the antibodies were compatible with either mouse or human (as applicable).

Eukaryotic cell lines

Policy information about cell lines

Cell line source(s)	B16F10 from ATCC CRL-6475; YUMM1.7 and YUMM3.1 were a gift from Marcus Bosenberg (Yale University, New Haven, CT).
Authentication	Cell lines were authenticated by morphology and pigment production (for B16F10).
Mycoplasma contamination	All cell lines tested negative for mycoplasma contamination.
Commonly misidentified lines (See ICLAC register)	N/A

Animals and other organisms

Policy information about studies involving animals; ARRIVE guidelines recommended for reporting animal research

Laboratory animals	C57BL/6, Ly5.1, Rag1-/-, Ldlr-/-, and B6.Cg-Aw-J EdaTa-6J +/+ ArTfm/J mice were purchased from Jackson Laboratory. 6-8 weeks old male and female mice were used in the experiments.
Wild animals	No wild animals were used in the study.
Field-collected samples	No field-collected samples were used.
Ethics oversight	Mice were housed in specific-pathogen-free conditions and cared for in accordance with US National Institutes of Health guidelines, and all procedures were approved by the Cedars-Sinai Animal Care and Use Committee.

Note that full information on the approval of the study protocol must also be provided in the manuscript.

Human research participants

Policy information about studies involving human research participants

Population characteristics	Patient ages ranged from 52-80 years (median 72). Races included: white (n=16), black (n=5), other (n=2), Asian (n=5), Hispanic (n=1), and American Indian (n=1). Patient samples were classified into two categories: patients not receiving any current treatment and patients undergoing active androgen deprivation therapy. Table S1 indicates clinical characteristics of each patient sample used in this study. a multivariable linear regression analysis with adjustments for presence of prostatectomy, age, race and current status was performed by the Biostatistics Core at Cedars Sinai. White blood cell count was not available for analysis and thus was not controlled for.
Recruitment	Patient recruitment was performed for this study under a separate protocol (Pro00042197). All prostate cancer patients entering the clinic were provided with the option to enroll in this single-center study. The investigators that received, processed, and analyzed these samples were blinded to the treatment group.
Ethics oversight	Blood specimens were studied under Cedars-Sinai Medical Center Institutional Review Board-approved protocol Pro00047412.

Note that full information on the approval of the study protocol must also be provided in the manuscript.

Flow Cytometry

Plots

Confirm that:

- [x] The axis labels state the marker and fluorochrome used (e.g. CD4-FITC).
- [x] The axis scales are clearly visible. Include numbers along axes only for bottom left plot of group (a 'group' is an analysis of identical markers).
- [x] All plots are contour plots with outliers or pseudocolor plots.
- [x] A numerical value for number of cells or percentage (with statistics) is provided.

Methodology

Sample preparation	Cell suspension was obtained from the lung or bone marrow. For bone marrow, the femur was snipped at both ends and was flushed using PBS. To obtain a single cell suspensions, the cells were passed through a 70µm cell strainer and red blood cells in cell pellets were lysed with ACK lysing buffer.<br>For lungs: The same lobe of the lung was manually digested in a lysis buffer containing HBSS, collagenase Type IV (2µg/mL;

	17104019; Thermo Fisher Scientific), and DNAseI (25 units/mL; 10104159001; Sigma-Aldrich) with two 10 minute incubations at 37°C. Samples were centrifuged for 5 min at 3000 RPM and the red blood cells were lysed using 1X RBC lysis buffer (eBioscience). Single cell suspensions were obtained from the digested lung using a 70 µm strainer.<br><br>To isolate murine neutrophils from the bone marrow or lungs, an EasySep Positive PE Selection Kit (19762A) or Negative Selection Mouse Neutrophil Enrichment Kit (18557) was used according to the manufacturer's protocols (Stemcell Technologies). The PE murine antibody used was Ly6G (1A8; BioLegend). Samples were pooled from 5 mice for each independent experiment and purity was confirmed if >93% by flow cytometry or by Siemens Diff-Quik Stain Set (B4132-1A, Fisher Scientific).<br><br>For human neutrophil isolation, peripheral blood mononuclear cells (PBMC) were removed using SepMate tubes and the Lymphoprep density gradient according to the manufacturer's protocol (85450;07801 StemCell Technologies). Once the PBMCs were removed, the pelleted blood was transferred to a new tube and the red blood cells were lysed using 1X RBC lysis buffer (eBioscience). Cells were stained using the Siemens Diff-Quik Stain Set to confirm >94% purity. Cells were maintained in Endothelial Basal Medium-2 (EBM2, CC-3156, Lonza) which was confirmed to not activate neutrophils.<br><br>Surface antigen staining was performed in PBS supplemented with 1% FBS for 20 minutes at 4ºC.
Instrument	Flow cytometry analysis was performed on a 13-colors LSR II (BD Biosciences).
Software	All events were acquired using BD FACS Diva software and data were analyzed with FlowJo software v.10 (TreeStar)
Cell population abundance	Purity of isolated neutrophils was confirmed to be greater than 94% by diff quick staining.
Gating strategy	Doublets were removed using Fs Lin vs FS Area.<br>Size exclusion was used to remove debris with FSC-area vs SSC-area.<br>T cells: CD4 or CD8 positive.<br>NK cells: NK1.1 positive, TCRbeta negative;b 49b positive for depletion studies.<br>NKT cells: NK1.1 positive, TCRbeta positive.<br>Neutrophils: CD11b positive, Ly6G positive, Gr-1 positive (for depletion studies).<br>Alveolar macrophages: CD11c positive, CD11b negative, F4/80 positive.<br>Dendritic cells: CD11c positive, CD11b positive, F4/80 positive.<br>Interstitial macrophages: CD11c negative, CD11b positive, F4/80 positive, Ly6G negative.<br>Human neutrophils: CD11b positive, CD66b positive.<br>MDSC-like human cells: CD11b positive, CD14 positive, HLA-DR negative, CD33 positive, CD15 positive.<br>The positive cells are indicated with a box in the supplementary figures.

☒ Tick this box to confirm that a figure exemplifying the gating strategy is provided in the Supplementary Information.
